# Temperature effects on synaptic transmission and neuronal function in the visual thalamus

**DOI:** 10.1371/journal.pone.0232451

**Published:** 2020-04-30

**Authors:** Matthew J. Van Hook

**Affiliations:** Department of Ophthalmology & Visual Sciences, Truhlsen Eye Institute, University of Nebraska Medical Center, Omaha, NE, United States of America; Doheny Eye Institute/UCLA, UNITED STATES

## Abstract

Numerous neuronal properties including the synaptic vesicle release process, neurotransmitter receptor complement, and postsynaptic ion channels are involved in transforming synaptic inputs into postsynaptic spiking. Temperature is a significant influencer of neuronal function and synaptic integration. Changing temperature can affect neuronal physiology in a diversity of ways depending on how it affects different members of the cell’s ion channel complement. Temperature’s effects on neuronal function are critical for pathological states such as fever, which can trigger seizure activity, but are also important in interpreting and comparing results of experiments conducted at room vs physiological temperature. The goal of this study was to examine the influence of temperature on synaptic properties and ion channel function in thalamocortical (TC) relay neurons in acute brain slices of the dorsal lateral geniculate nucleus, a key synaptic target of retinal ganglion cells in the thalamus. Warming the superfusate in patch clamp experiments with acutely-prepared brain slices led to an overall inhibition of synaptically-driven spiking behavior in TC neurons in response to a retinal ganglion cell spike train. Further study revealed that this was associated with an increase in presynaptic synaptic vesicle release probability and synaptic depression and altered passive and active membrane properties. Additionally, warming the superfusate triggered activation of an inwardly rectifying potassium current and altered the voltage-dependence of voltage-gated Na^+^ currents and T-type calcium currents. This study highlights the importance of careful temperature control in ex vivo physiological experiments and illustrates how numerous properties such as synaptic inputs, active conductances, and passive membrane properties converge to determine spike output.

## Introduction

Although temperature is understood to be an important factor regulating neuronal excitability and synaptic transmission, it is not known how temperature affects the multiple processes involved in turning strong sensory-driven synaptic inputs into spike outputs in thalamic relay neurons. Different ion channel types are regulated in different ways by altered temperature and this complex set of changes can have unpredictable effects on neuronal response properties [1–8]. Thus, understanding how temperature regulates synaptic inputs and generation of output spiking in distinct neuron classes is important for translating findings from reduced *ex vivo* preparations to understanding neuronal function *in vivo*. In cold-blooded species, different synaptic and intrinsic neuronal processes might function to balance each other in changing temperature environments so that function remains relatively stable in the face of dramatically variable temperature conditions [9]. In mammals, however, loss of temperature homeostasis, as occurs in hypo- or hyperthermia can have pathological effects on neuronal function, as occurs in febrile seizures in hyperthermia [10]. Experiments in human subjects have also shown that modest changes in body temperature appear able to alter visually-evoked potentials [11] and lowering body temperature has a neuroprotective effect on the survival and function of retinal neurons in a model of optic nerve injury [12].

The dorsal lateral geniculate nucleus (dLGN) of the thalamus is a key subcortical projection target for retinal ganglion cells (RGCs), the output neurons of the retina [13,14]. RGCs make excitatory synapses onto dLGN thalamocortical (TC) relay neurons and this strong “driver” input from RGCs is integrated by the passive membrane properties and active conductances in TC neurons to trigger post-synaptic spiking output that is relayed predominantly to layer IV of the primary visual cortex. Notably, TC neurons operate in either burst or tonic spiking modes that are regulated strongly by resting membrane potential and the consequent inactivation state of T-type Ca^2+^ channels and firing mode appears to be strongly regulated by non-retinal synaptic and neuromodulatory inputs [2,15–18]. TC neuron hyperpolarization facilitates bursting behavior by relieving T-type current inactivation so that depolarization triggers a low-threshold Ca^2+^ spike and voltage-gated Na^+^ channel-dependent action potentials riding atop it. In contrast, slight depolarization leads to T-type channel inactivation, facilitating tonic firing. As a result, while tonic mode supports a more linear integration of retinal inputs, burst mode appears to favor a non-linear encoding of visual information [19–23]. Currents flowing through both voltage-gated Na^+^ channels and T-type Ca^2+^ channels are known to be altered by temperature [1,2,4,6].

The goal of this study was to determine how temperature affects RGC-driven synaptic input and spiking output of TC neurons in the dLGN and to probe how key membrane currents responsible for shaping TC neuron spike output are modulated by temperature. As a result, these experiments focused on temperature regulation of presynaptic vesicle release properties as well as voltage-gated Na^+^ currents, T-type Ca^2+^ currents, and an inwardly-rectifying potassium current (Kir). Kir has not previously been described in dLGN TC neurons. This was accomplished using an optogenetic reporter mouse line in combination with whole-cell patch clamp recordings in acute dLGN brain slices.

## Materials and methods

Animal protocols were approved by the Institutional Animal Care and Use Committee at the University of Nebraska Medical Center. C57Bl/6J and Chx10-Cre;Ai32 mice of both sexes were housed on a standard 12:12 hour light:dark cycle and provided with food *ad libitum*. Mice were 5–10 weeks old at time of experiments. Chx10-Cre;Ai32 mice were generated as a cross of Chx10-Cre mice [24] and Ai32 mice [25], which allows for expression of a channelrhodopsin-2 (ChR2)/yellow fluorescent protein (YFP) fusion protein in retinal ganglion cell axons projecting to the dLGN [26]. These mice were used for studies of temperature’s effect on retinogeniculate synaptic transmission, while C57Bl/6J mice were used for all other experiments.

250 micron-thick coronal brain slices containing the dLGN were prepared using the “protected recovery” approach [27,28], as described previously [29]. Following euthanasia with CO_2_ and cervical dislocation, mice were decapitated and brains were dissected into a slush of ice-cold artificial cerebrospinal fluid (aCSF) composed of (in mM) 128 NaCl, 2.5 KCl, 1.25 NaH_2_PO_4_, 24 NaHCO_3_, 12.5 glucose, 2 CaCl_2_, 2 MgSO_4_ and bubbled with 5% CO_2_ in 95% O_2_. The cerebellum was removed with a razor blade and the brain was affixed to the stage of a Leica VT1000S vibratome with cyanoacrylate glue. Slices were cut in ice-cold aCSF using a stainless steel razor blade. After being hemisected along the midline they were transferred to a nylon net in a beaker containing an NMDG-based solution (in mM: 92 NMDG, 2.5 KCl, 1.25 NaH_2_PO_4_, 25 glucose, 30 NaHCO_3_, 20 HEPES, 0.5 CaCl_2_, 10 MgSO_4_, 2 thiourea, 5 L-ascorbic acid, and 3 Na-pyruvate) bubbled with 5% CO_2_ in 95% O_2_ and warmed to ~30°C. After incubating in the NMDG solution for 10 minutes, slices were transferred to a nylon net in a beaker containing room temperature aCSF and allowed to recover for >1 hour prior to patch clamp experiments.

For electrophysiology experiments, slices were positioned in a recording chamber on an upright fixed-stage microscope (Olympus BX51WI) and superfused with aCSF supplemented with 60 μM picrotoxin at ~2–4 mL/min. Temperature was varied from room temperature (~22–24°C) to warmed bath conditions (~31–34°C) using an in-line solution heater (Warner Instruments) and measured in the bath near the slice. Thalamocortical (TC) relay neurons in the dLGN were targeted for whole-cell voltage- or current-clamp recording using patch pipettes pulled from thin-walled borosilicate tubing with an internal filament. A Multiclamp 700A amplifier, DigiData 1550B A-D/D-A interface, and pClamp 10 electrophysiology software (Axon/Molecular Devices) were used for data acquisition. A K-gluconate pipette solution was used for current-clamp and voltage-clamp recordings of inward-rectifying K^+^ currents and mEPSCs: (in mM) 120 K-gluconate, 8 KCl, 2 EGTA, 10 HEPES, 5 ATP-Mg, 0.5 GTP-Na_2_, 5 phosphocreatine (pH = 7.4, 275 mOsm). A Cs-methanesulfonate pipette solution was used for voltage-clamp recordings of retinogeniculate synaptic transmission and T-type Ca^2+^ currents: 120 Cs-methanesulfonate, 2 EGTA, 10 HEPES, 8 TEA-Cl, 5 ATP-Mg, 0.5 GTP-Na_2_, 5 phosphocreatine-Na_2_, 2 QX-314 (pH = 7.4, 275 mOsm). The Cs-based solution was also used for recording voltage-gated Na^+^ currents, although the QX-314 was omitted for those experiments. For recordings of sodium currents, the aCSF bathing solution was supplemented with 60 μM picrotoxin, 500 μM BaCl_2_, 20 μM CNQX, and 100 μM CdCl_2_. Reported voltages are corrected for a -10 or -14 mV liquid junction potential for the Cs- and K-based pipette solutions, respectively.

Optogenetic stimulation of ChR2-expressing RGC axons was accomplished with 460 nm full field flashes (0.5 ms) generated by a TLED system (Sutter Instruments) and delivered through the microscope objective. The TLED power, calibrated using a Metrologic digital laser power meter, was 1.5 mW. Paired pulse experiments were performed by varying the interval between stimuli (100 ms to 1 s interval). Release probability was measured using a linear fit to the cumulative EPSC amplitudes of the last 15 responses to a 3-s duration 10-Hz train stimulus with the TLED. The RGC spike train stimulus was generated using a cell-attached recording of spiking behavior from an On-sustained αRGC.

For measurements of short-term synaptic plasticity and retinogeniculate synaptic vesicle release probability, the bath was supplemented with 200 μM γD-glutamylglycine (γDGG, Abcam, Cambridge, MA) and 100 μM cyclothiazide (Santa Cruz, Dallas, TX) [30]. 500 μM BaCl_2_ was added to the bath solution when indicated in order to block Kir.

mEPSCs were recorded in a 60-second duration recording in the absence of stimulation at a holding potential of -74 mV. For each cell, the first ~100 events were detected and analyzed using MiniAnalysis software (Synaptosoft Inc, Fort Lee, NJ). Using the same number of events for each recorded cell avoids biasing the cumulative distribution analysis of event amplitude and inter-event intervals.

Series resistance was monitored for all cells and compensated by 60–65% during recordings of EPSCs in slices from Chx10-Cre;Ai32 mice and for recordings of voltage-gated Na^+^ currents, T-type Ca^2+^ currents, and Kir. The bridge balance circuitry was used to compensate for series resistance in current clamp recordings. Data were excluded from analysis if series resistance changed dramatically (>30%) during the course of the recording.

Temperature coefficient (*Q10*) values were calculated using the following formula:

$$Q10={\left(\frac{R2}{R1}\right)}^{\left(\frac{10}{T2-T1}\right)}$$

Where, *R1* and *R2* are the measured parameter in room temp and warmed conditions, respectively, while *T1* and *T2* are measured temperature at room temperature and warmed conditions, respectively.

For current clamp experiments, spikes were detected using the “event detection” function of Clampfit. Spike frequency adaptation was assessed using a spike adaptation index (SAi), which was the ratio of average intervals of the first three action potentials to the average of the action potential intervals at the end of the current step. SAi = 1 means that the spike frequency does not change during a stimulus. SAi<1 indicates that spike frequency slows while SAi>1 is indicative of accelerating spike frequency. Input resistance was measured as the slope of a straight line fit to voltage deflection amplitudes evoked by current stimuli of -20 and -40 pA.

Statistical analyses were performed in GraphPad Prism 7 or Clampex 10 software. Normality of the data distributions was tested using the D’Agnostino-Pearson omnibus normality test in GraphPad Prism 7. Significance was tested using a two-tailed paired t-test for normally-distributed data and a Wilcoxon matched pairs signed rank test for non-normally-distributed data, as indicated. Differences in cumulative distributions of mEPSC intervals and amplitudes were assessed using a Komolgorov-Smirnov (K-S) test. P<0.05 was considered statistically significant for t-test and Wilcoxon matched pairs signed rank tests and p<0.0005 for the Komolgorov-Smirnov test. Data are presented as mean ± SEM unless indicated otherwise and sample sizes refer to the number of cells.

## Results

**Fig 1:**
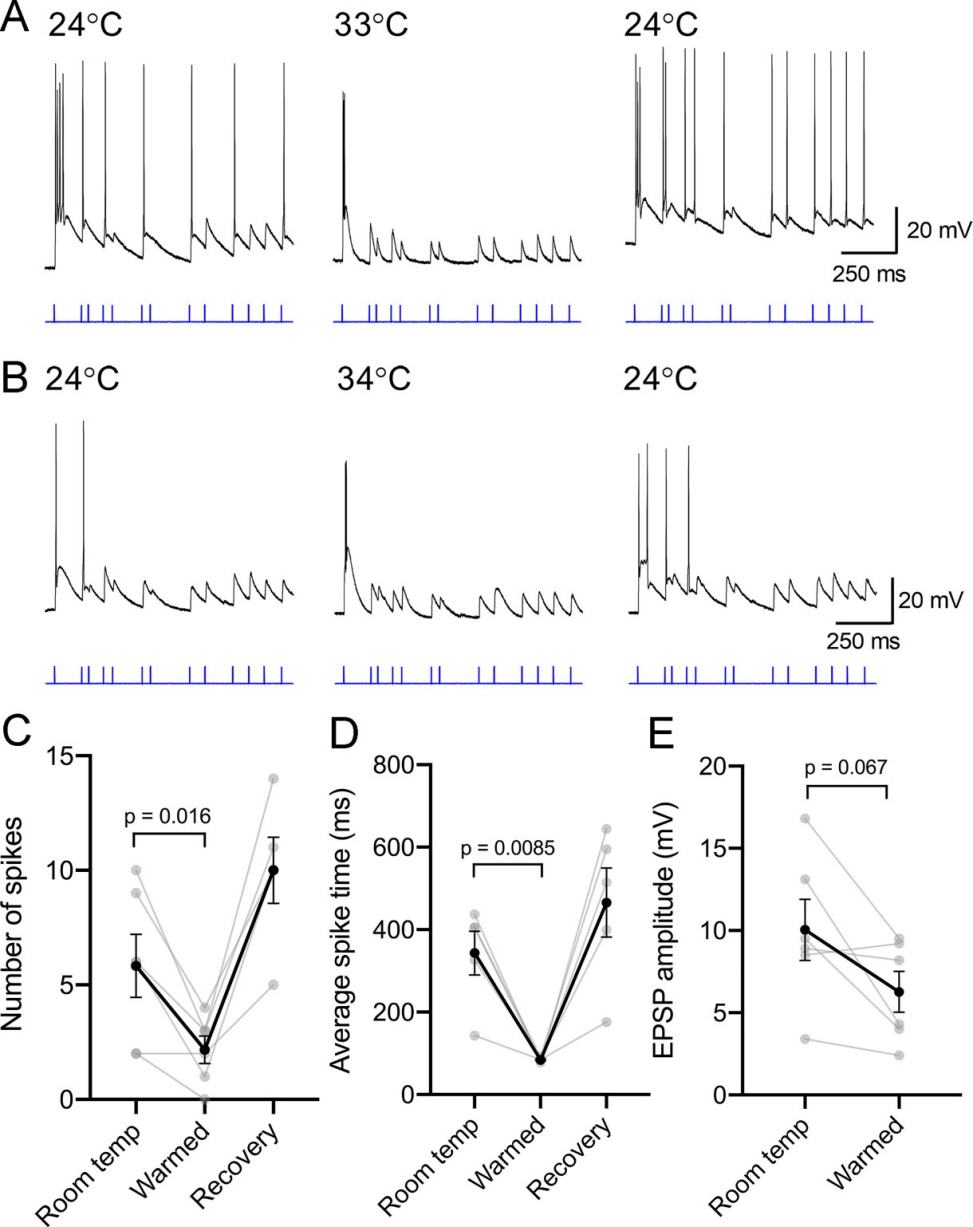
Warming reduces the spiking responses of dLGN thalamocortical relay neurons to optic tract stimulation. A & B) Whole-cell current clamp recordings of two example dLGN TC neurons from Chx10-Cre;Ai32 mice in which a 460 nm LED was used to deliver a stimulus sequence (blue) derived from a retinal ganglion cell spike train. Warming ultimately reduced the responses of the TC neurons, concentrating spiking to the first stimulus flash. C) The number of spikes evoked during the stimulus sequence was reduced by warming the bath and recovered following return to room temperature conditions. D) The average of the peak time of each spike in the stimulus sequence was used to examine the distribution of spikes over the sequence. The average peak time shifted to smaller values in warmed conditions, indicating that spikes were more concentrated to the beginning of the stimulus sequence. E) The amplitude of sub-spike threshold EPSPs at a point in the sequence that reliably did not evoke a spike was measured in each of the six cells and was slightly reduced by warming, although the change was not statistically significant.

Studies of neuronal function in mammalian brain tissue are occasionally conducted at temperatures lower than physiological body temperature. In order to determine how temperature affects the integration of synaptic inputs and the spike generation mechanism(s) in TC neurons in the mouse dLGN, RGC axons in coronal slices from Chx10-Cre;Ai32 mice were stimulated with a 460 nm LED flash while synaptically-driven spiking behavior was recorded from TC neurons at room temperature (~23–24°C) and in warmed conditions (~33°C) (Fig 1, S1 Fig). In order to somewhat mimic RGC input to dLGN TC neurons, slices were stimulated with a train of 460 nm LED flashes generated from the spiking behavior of an On-sustained αRGC. Multiple RGC types project to the dLGN and so the precise pattern of presynaptic input will surely vary depending on the presynaptic RGC class, visual scene, etc. Thus, although not necessarily representative of the precise inputs a given TC neuron might receive, this stimulus paradigm was chosen to represent a closer-to-physiological pattern of presynaptic stimulation since it consists of a mix of inter-stimulus intervals derived from the spike train of an actual RGC.

Using the RGC-derived stimulus sequence, there was variability in the synaptically-driven spiking responses across the population of six sampled TC neurons; at room temperature, some robustly fired action potentials over the course of the stimulus sequence, while others generated only a few, which were typically concentrated to the beginning of the sequence. Increased temperature is generally thought to enhance neuronal responsiveness and excitability by upregulating a variety of synaptic and neuronal functions [10]. Surprisingly, however, warming the bath universally reduced TC spiking occurring over the course of the stimulus sequence. This was quantified by counting the number of action potentials fired during the stimulus sequence (Fig 1C). The action potential number was reduced from 5.8 ± 1.4 to 2.2 ± 0.6 (n = 6, p = 0.016. Action potential firing recovered when the bath temperature was allowed to cool back to approx. 24°C. Additionally, although the first stimulus in the sequence generated a robust response in warmed conditions (in some cases, the number of spikes evoked on the first stimulus was increased), spiking responses to later stimuli in the sequence were diminished. This was quantified by averaging the time at which all recorded spikes occurred during the ~1.25 second long stimulus sequence (Fig 1D). Lower values point to a concentration of spikes occurring at the beginning, while higher values reflect spikes occurring throughout the sequence. With warming, this value was reduced from 343 ± 53 ms to 85 ± 2 ms (n = 5; p = 0.0085). One cell that did not fire any action potentials in warmed conditions was excluded from this analysis.

In many of the recorded cells, it appeared that sub-spike threshold excitatory post synaptic potentials (EPSPs) were smaller in warmed conditions than at room temperature. To test this, the amplitude of an EPSP that occurred approx. 100 ms following the previous stimulus flash and generally did not reach spike threshold was measured (Fig 1E). Although the EPSP amplitude was slightly reduced (10.0 + 1.9 mV to 6.3 + 1.2 mV), this change was not statistically significant (p = 0.067 n = 6).

**Fig 2:**
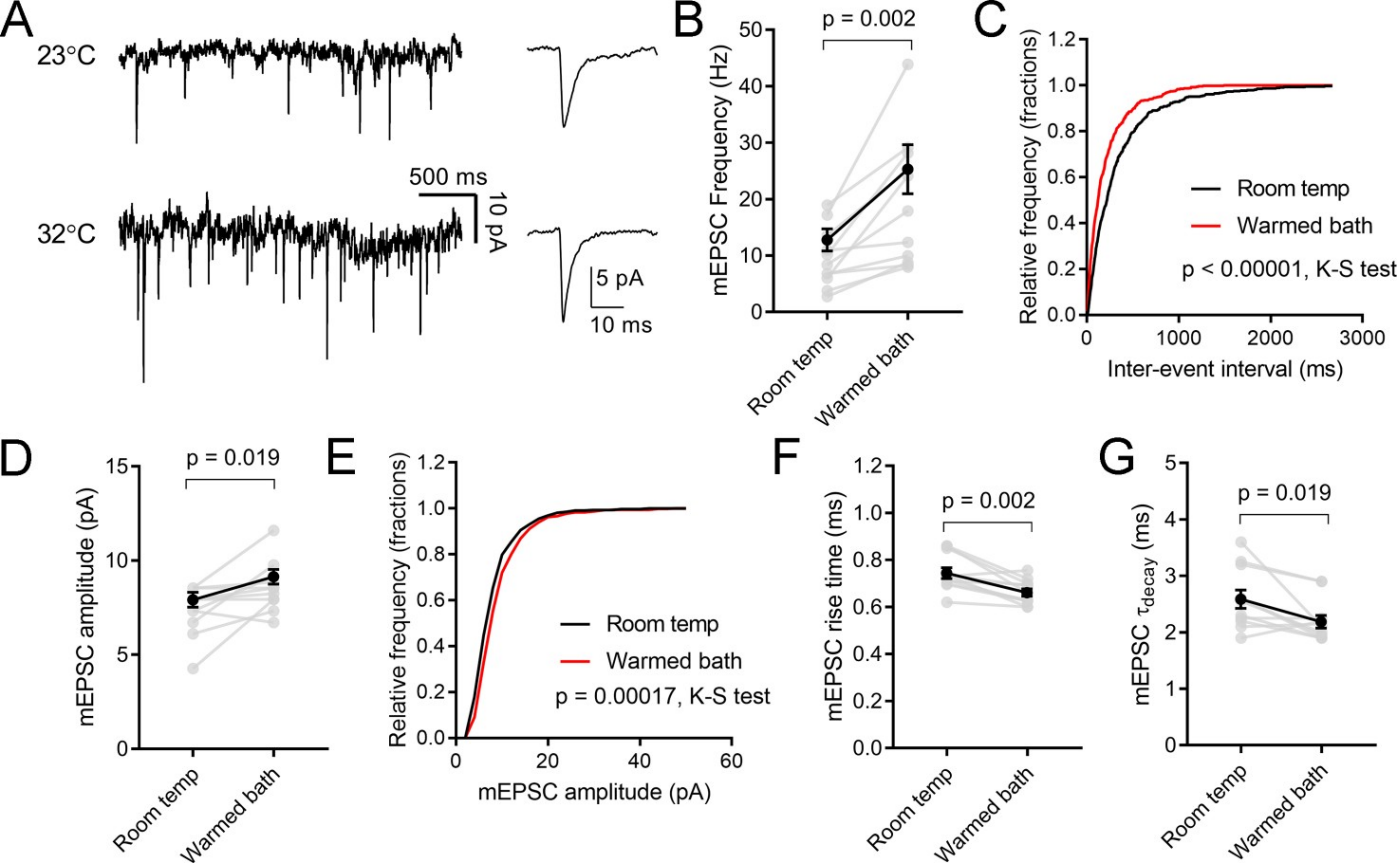
Temperature alters mEPSCs recorded in dLGN TC neurons. A) Voltage-clamp traces of mEPSCs recorded in the absence of stimulation at room temperature and then following warming of the bath solution. Traces on the right show the average mEPSC waveforms detected in each temperature condition from that cell. B) Median mEPSC frequency from 11 TC neurons increased with warming. C) Cumulative distribution of mEPSC inter-event intervals (100 events per temperature condition per recorded cell) show that the intervals shifted left indicating that frequency increased. D) Plots of median mEPSC amplitude from 11 TC neurons show that mEPSC amplitude increased with warming. E) Cumulative distribution of mEPSC amplitudes. F & G) Warming accelerated the mEPSC rise time and decay time constant.

**Fig 3:**
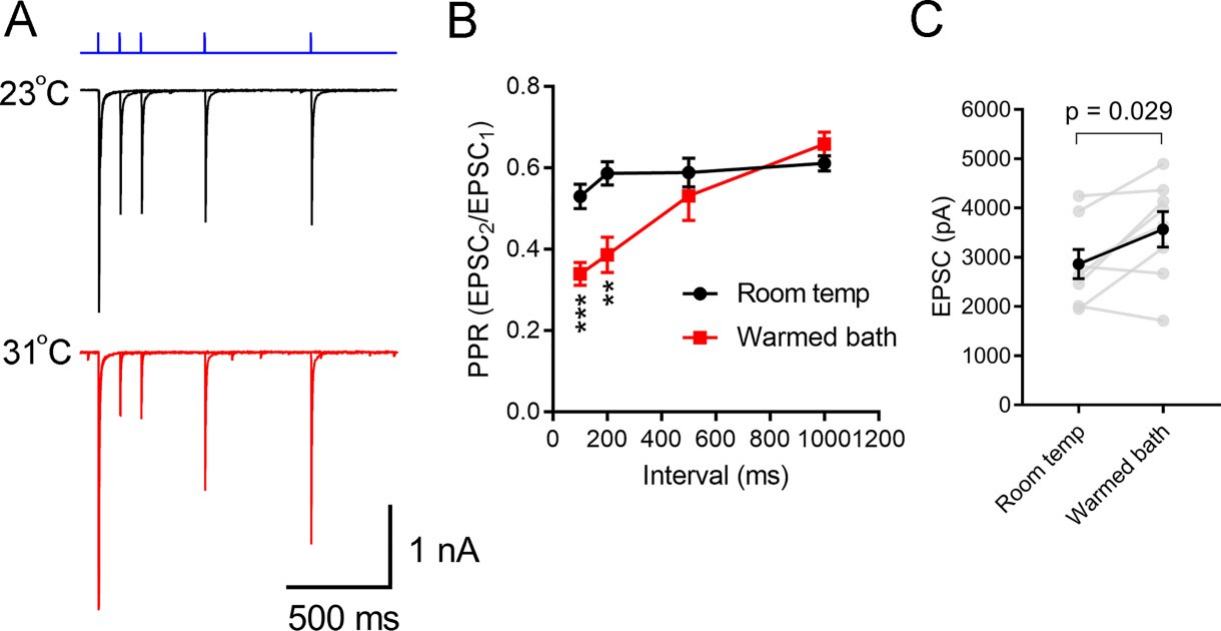
Influence of temperature on short-term plasticity of retinogeniculate synapses. A) Overlaid voltage-clamp recordings from a TC neuron from a Chx10-Cre;Ai32 mouse in response to pairs of 460 nm LED stimuli with intervals of 100, 200, 500, and 1000 ms (blue) at 23°C and 31°C in the presence of 200 μM γDGG and 100 μM cyclothiazide. Recordings were made in the presence of γDGG (200 μM) and cyclothiazide (100 μM). B) Plot of paired pulse ratio (PPR = EPSC2/EPSC1) showing that synaptic depression was increased following warming of the bath solution (n = 6). **p<0.01; ***p<0.001, t-test. C) Measurements of EPSC amplitude at room temperature and in warmed conditions (n = 8).

The convergence of multiple parameters including synaptic inputs (quantal content, vesicle pool size, synaptic release probability), passive membrane properties (membrane input resistance, time constant), and active membrane properties (voltage-gated currents and spike generation) determines neuronal spike output in response to presynaptic stimulation. To test whether/how altered synaptic inputs and changed short-term synaptic plasticity might contribute to this observed effect, miniature excitatory post-synaptic currents (mEPSCs) were recorded in whole-cell voltage-clamp recordings from TC neurons in the absence of stimulation (Fig 2). mEPSC instantaneous frequency recorded in n = 11 TC neurons was 9.6 ± 1.6 Hz at room temperature and increased to 19.6 ± 3.5 Hz in warmed bath conditions (p = 0.0021, t-test; Fig 2A and 2B). An analysis of the cumulative distribution of mEPSC inter-event intervals (100 mEPSCs per recorded cell at room temperature and 100 mEPSCs in warmed bath conditions) showed a significant shift in the population distribution to shorter inter-event intervals (p<0.0005; K-S test; Fig 3C). This increase in mEPSC frequency was accompanied by a small, but statistically significant, increase in mEPSC median amplitude (7.4 ± 0.4 pA to 8.6 ± 0.4 pA; p = 0.02, Wilcoxon matched-pairs test; p < 0.0005, K-S test; Fig 2D & 2E) as well as an acceleration of mEPSC rise and decay kinetics (rise time: 0.75 ± 0.02 ms to 0.66 ± 0.02 ns; p = 0.002; τ_decay_: 2.6 ± 0.16 ms to 2.2 ± 0.11 ms, p = 0.019; Fig 2F & 2G). An increase in mEPSC amplitude might point to higher AMPA receptor single channel conductance, while the increased mEPSC frequency would be consistent with an increase in Pr.

To quantitatively test for changes in Pr, two approaches were employed where presynaptic retinal ganglion cell axons were optogenetically stimulated in brain slices from Chx10-Cre;Ai32 mice. Flashes delivered through the objective lens from a 460 nm LED evoked robust excitatory post-synaptic currents (EPSCs) in voltage-clamped TC neurons. These recordings were performed in the presence of γDGG and cyclothiazide to prevent AMPA receptor saturation and desensitization, respectively. In the first approach, a paired pulse experiment, RGC axons were stimulated with pairs of flashes separated by 100 to 1000 ms (Fig 3A). In room temperature recordings, synapses displayed short-term synaptic depression, with the second response being smaller than the first across all inter-stimulus intervals tested so that the paired pulse ratio (PPR = EPSC2/EPSC1) was <1 (Fig 3B). This is typical of synapses with a high probability of synaptic vesicle release (Pr) and has been previously documented in studies of retinogeniculate synaptic function [26,29,31,32]. When the bath was warmed, synaptic depression became even more pronounced, with the PPR being reduced relative to room temperature conditions. At a 100 ms interval, for instance, PPR was reduced from 0.53 ± 0.03 to 0.34 ± 0.03; n = 6; p < 0.001). The PPR recovered when the bath was returned to room temperature. Additionally, warming increased EPSC amplitudes, from 2865 ± 294 pA to 3571 ± 360 pA (n = 7, p = 0.029; Fig 3C). The Q10 for the EPSC amplitudes was 1.3 ± 0.1. EPSC amplitude recovered when the bath was returned to room temperature. The decreased PPR and increased EPSC amplitude accompanying warming are consistent with an increase in Pr [30].

**Fig 4:**
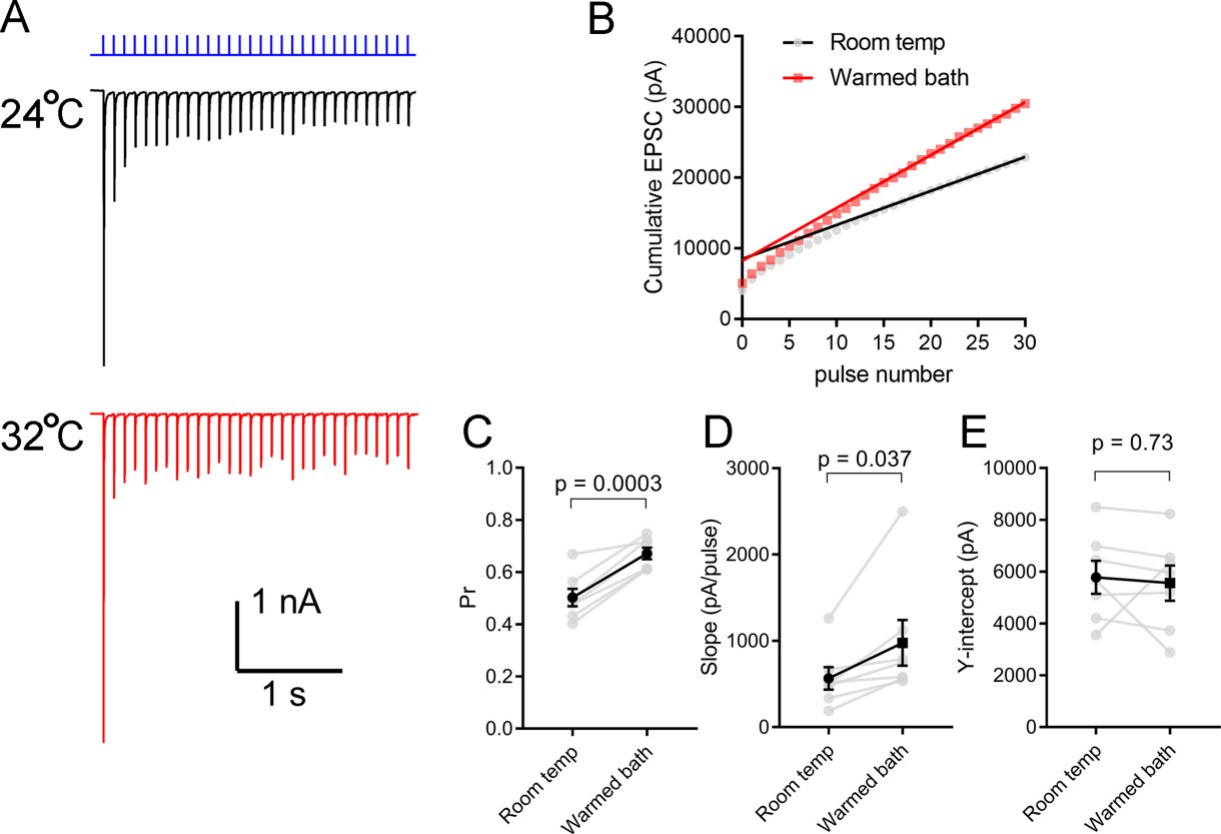
Temperature regulates synaptic vesicle release probability and replenishment at retinogeniculate synapses. A) Voltage-clamp recordings from a dLGN TC neuron EPSCs in response to a 10 Hz stimulation of retinal ganglion cell axons using a 460 nm LED in a brain slice from a Chx10-Cre;Ai32 mouse. Recordings were made in the presence of γDGG (200 μM) and cyclothiazide (100 μM). Each trace is the average of 3–5 individual responses to the same stimulus. B) Plot of cumulative EPSC amplitude and linear fit to the responses at pulses 15–30. Extrapolation to the Y-intercept is used as a measure of vesicle pool size while the slope is a measure of replenishment rate. Release probability is calculated as the ratio of the first EPSC to the Y-intercept. C) Vesicle release probability increased with warming the bath solution (n = 7 TC neuron recordings). D) Vesicle pool replenishment rate (slope of the fit in panel B) also increased with warming. E) The Y-intercept, which is a measure of vesicle pool size was not changed by warming.

Next, using a complementary stimulation paradigm, dLGN slices from Chx10-Cre;Ai32 mice were stimulated with a three second-long train of LED stimuli delivered at 10 Hz (Fig 4A). After partial depletion of the synaptic vesicle pool, the rate of continued synaptic vesicle release is limited by the rate of replenishment at the synapse. In this analysis, Pr was measured as the ratio of the first EPSC amplitude to the Y-intercept of a straight line fit to responses 15–30 of the cumulative EPSC amplitude (Fig 4B) [30]. Consistent with the paired pulse data, Pr measured in this way increased from 0.50 ± 0.03 to 0.67 ± 0.02 (n = 7, p < 0.001; Fig 4C). The Q10 for Pr was 1.4 ± 0.1.

In these recordings, changes in the slope of the fit to the steady-state cumulative EPSC can serve as a relative measure of synaptic vesicle replenishment to the synapse and physiological temperature has been shown at the calyx of Held to accelerate vesicle recruitment [33]. When the bath was warmed, the slope of this fit increased from 565 ± 129 pA/pulse to 977 ± 265 pA/pulse (p = 0.037; Fig 4D), suggesting that replenishment rate was increased at warmer temperatures, although the small increase in quantal amplitude likely makes a small contribution to this difference as well. However, raising the temperature did not appear to cause a change in synaptic vesicle pool size, as the Y-intercept of the fit to the steady-state did not significantly change following warming (5780 ± 639 pA at room temp and 5556 ± 682 pA in warmed conditions, p = 0.73; Fig 4E). This mirrors effects at some synapses [34], but contrasts with temperature effects at others [35–37].

Thus, the concentration of synaptically-driven spiking to the beginning of the RGC stimulus sequence in warmed conditions might be partially attributable to an increase in synaptic vesicle release probability and consequent increase in short-term synaptic depression so that fewer vesicles are available at later pulses to drive post-synaptic spiking.

**Fig 5:**
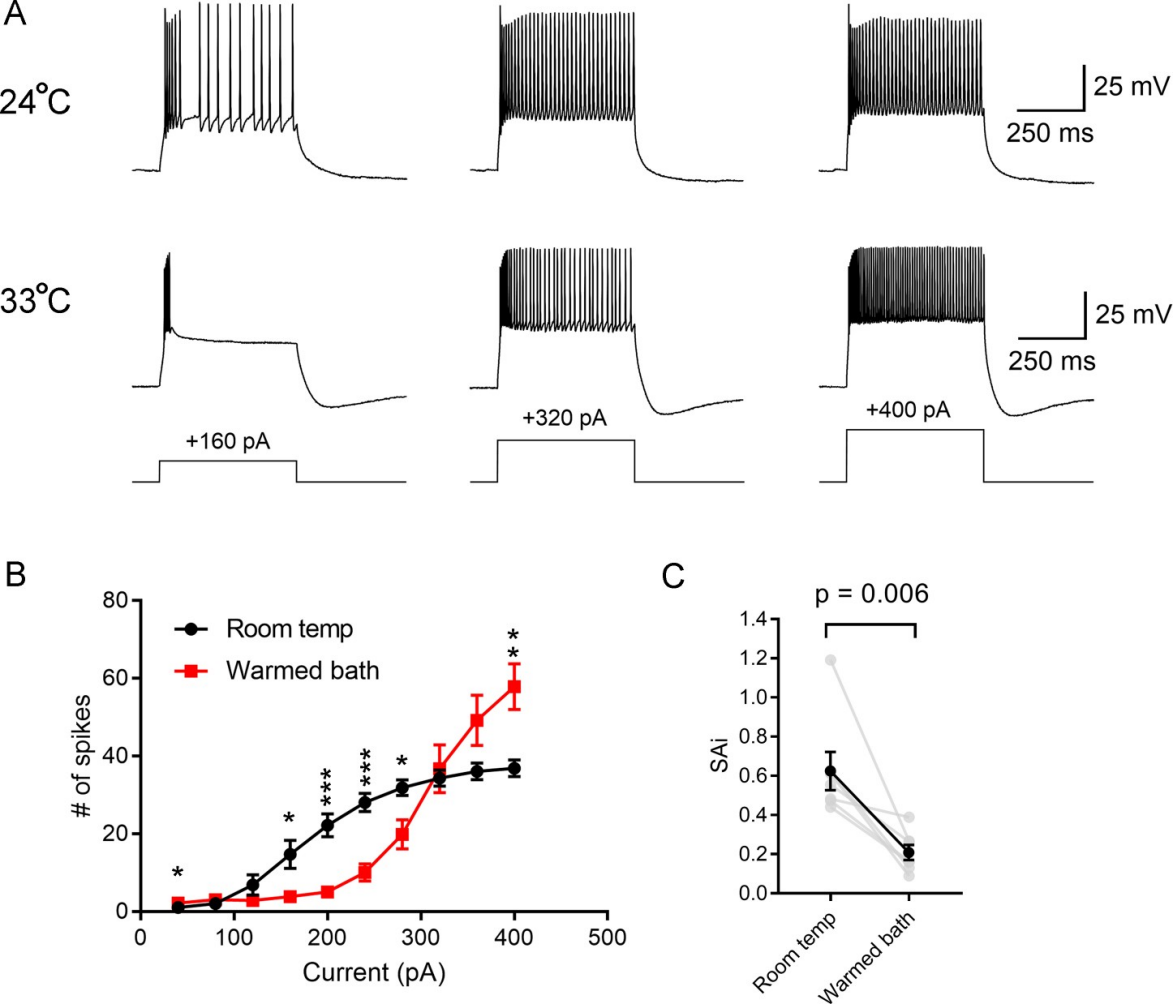
Temperature regulates the excitability of dLGN TC neurons. A) Current clamp recordings of TC neurons in response to 500-ms depolarizing current injections at room temp (24°C) and in warmed conditions (33°C). Warming reduced the number of evoked spikes at the weaker stimulus (+160 pA), but increased spiking at stronger stimuli (+400 pA). B) Plot of the number of evoked spikes during a 500 ms current injection in room temperature and warmed conditions show that warming reduced the number of evoked spikes at lower current stimulus strengths, but increased spiking at higher stimulus strengths. *p<0.05, **p<0.01, ***p<0.001. C) Temperature regulates spike frequency adaptation. The spike adaptation index (SAi), which was the ratio of the average intervals of the first three spikes to the average intervals at the end of the stimulus, was measured at the +320 pA stimulus as this evoked a similar number of action potentials at room temperature and in warmed conditions. The reduction in SAi indicates that spike adaptation was enhanced by warming.

To test whether spike generation itself was altered, TC neuron spiking behavior was measured in response to 500-ms long pulses of depolarizing current stimuli (40–400 pA, 40 pA intervals) in whole-cell current clamp recordings at room temperature and in warmed bath conditions (Fig 5A). Strikingly, warming the bath solution led to a decrease in the number of action potentials evoked during the 500 ms stimulus at current stimuli up to approx. 320 pA. Stimuli stronger than 320 pA, however, evoked a greater number of action potentials (n = 7; Fig 5B).

TC neurons are characterized by a notable spike frequency adaptation [16,38], where the spike rate slows during a sustained depolarizing stimulus. This phenomenon contributes to bursting/phasic spiking behavior of TC neurons and is the result of both LVA Ca^2+^ currents and their ability generate a low-threshold Ca^2+^ spike at the beginning of a stimulus as well as Ca^2+^-activated K^+^ and Cl^-^ currents that repolarize the membrane [16,38–40]. Warming the bath also led to a pronounced increase in spike frequency adaptation. To quantify this, a spike adaptation index (SAi) was calculated as the ratio of early to late inter-spike intervals for spikes evoked in response to a 500-ms duration 320 pA current injection stimulus. This stimulus was chosen for this analysis as it generated a similar number of action potentials in both room temperature and warmed conditions. A SAi = 1 indicates no adaptation while SAi<1 is indicative of adaptation and SAi>1 of an acceleration of spike frequency. Warming the bath led to a decrease in SAi from 0.62 ± 0.1 to 0.21 ± 0.04 (n = 7; p = 0.0061; Fig 5C), meaning that spike adaptation was more pronounced.

**Fig 6:**
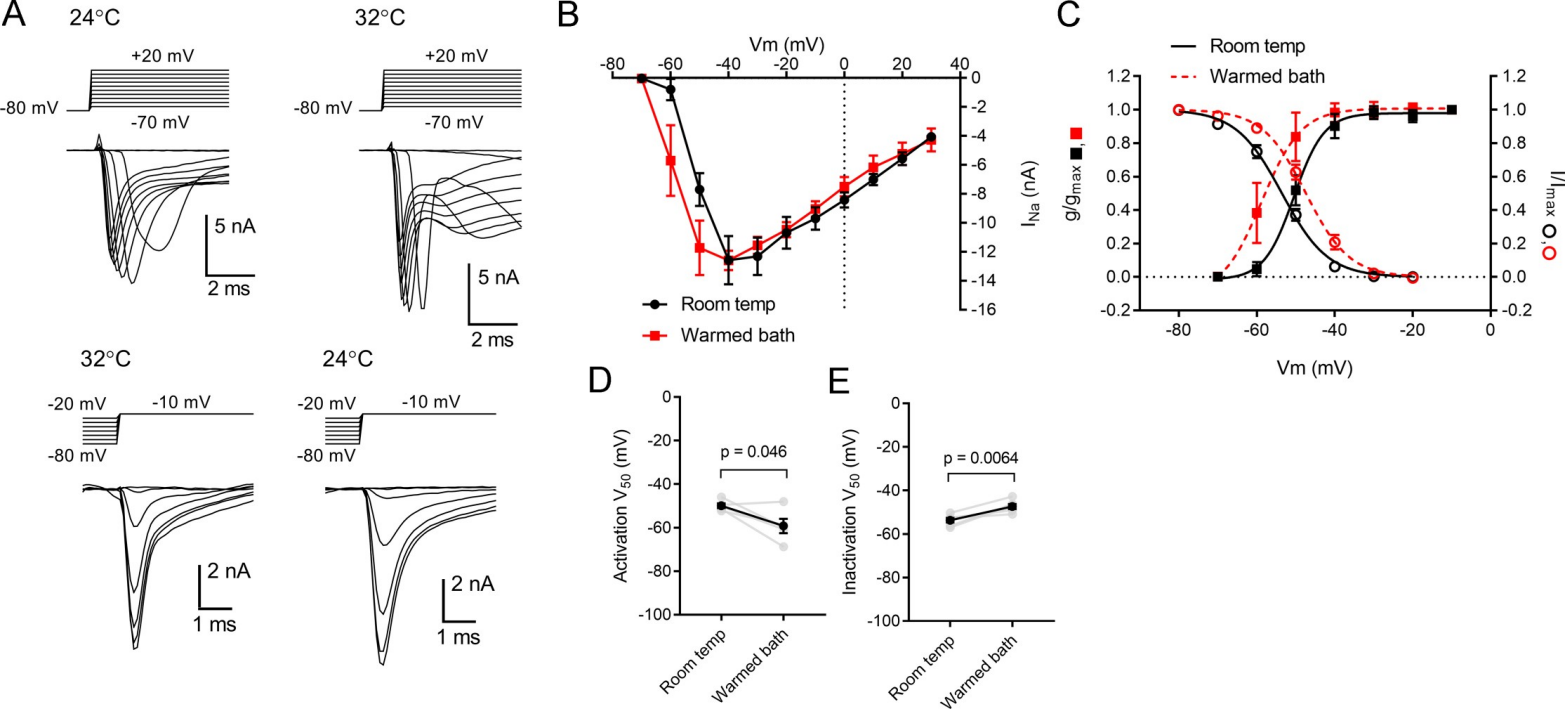
Temperature regulates the activation and steady-state inactivation of voltage-gated sodium currents in dLGN TC neurons. A) Whole-cell voltage clamp recordings of I_Na_ activation (top) and inactivation (below) in room temperature and warmed conditions. The displayed activation and inactivation data were obtained from two different cells. For the displayed inactivation experiment, the room temperature measurements were obtained after the bath returned to 24°C. The bathing solution was supplemented with 60 μM picrotoxin, 500 μM BaCl_2_, 20 μM CNQX, and 100 μM CdCl_2_. B) The I-V plot of I_Na_ shows that current amplitude did not significantly increase (p>0.05 for data points at each voltage), although it appeared to shift its voltage-dependence to more hyperpolarized voltages. C) Plot of g_Na_ activation and I_Na_ steady-state inactivation shows a leftward (hyperpolarized) shift of activation and a rightward (depolarized) shift in inactivation. D & E) V50 values for activation (N = 5) and inactivation (N = 6) are plotted for room temperature and warmed conditions.

Finding that warming leads to altered spiking behavior, voltage-clamp experiments were undertaken to probe for alterations in ion channel properties in TC neurons. To test whether temperature affects voltage-gated Na^+^ channel function, TC neurons were voltage-clamped at -80 mV and depolarized with a series of steps at 10 mV increments (Fig 6A). Warming the bath solution led to a small hyperpolarizing shift in the I_Na_ current-voltage plot (n = 5 TC neurons; Fig 6B), although I_Na_ amplitude values were not significantly different at each voltage. When Na^+^ current conductance (g) was calculated by correcting for driving force and the g/g_max_ was fit with a Boltzmann sigmoidal, the V50 was hyperpolarized by 9.3 ± 3.3 mV (n = 5) in warmed conditions compared to room temperature (V50 room temp = -49.9 ± 1.1 mV; warmed = -59.2 ± 3.3; p = 0.046; Q10 = 1.21 ± 0.08; Fig 6C & 6D). Consistent with previous reports [6,41,42], warmed temperatures also led to a depolarizing shift in the voltage-dependence of I_Na_ steady-state inactivation (Fig 6A). To measure this, TC neurons were depolarized for 150 ms to several test potentials in 10 mV increments from a holding potential of -90 mV before a 50-ms depolarization to -10 mV to fully activate I_Na_. Fits of the average I/I_max_ revealed a depolarizing shift in I_Na_ steady-state inactivation, from a V50 of -53.6 ± 1.0 mV at room temperature to -47.3 ± 1.1 mV in warmed conditions (6.3 ± 1.4 mV shift, n = 6 TC neurons; p = 0.0065; Q10 = 1.15 ± 0.03; Fig 6C & 6E).

**Fig 7:**
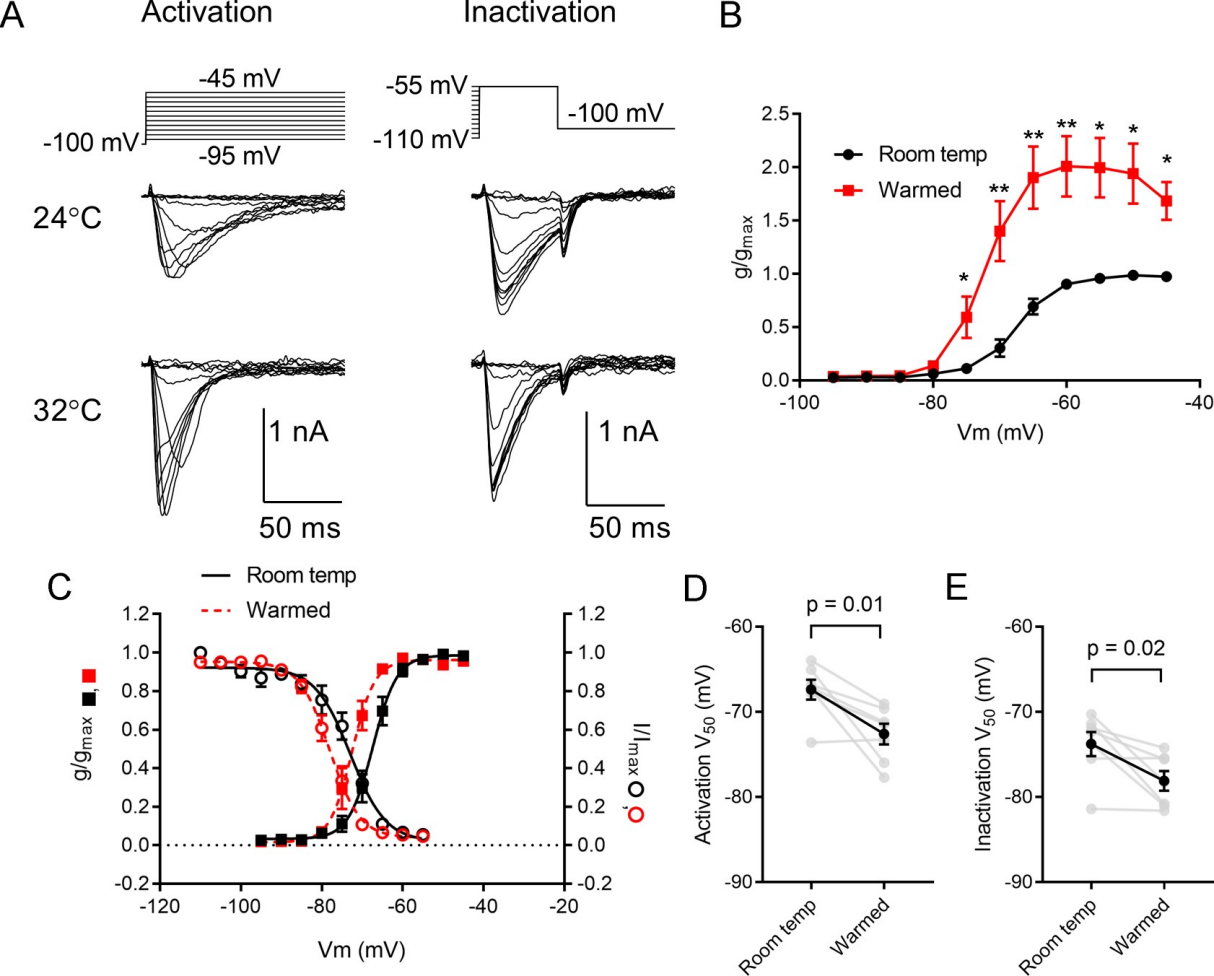
Temperature regulates the activation and inactivation of T-type LVA Ca^2+^ currents in dLGN TC neurons. A) Recordings of I_Ca_ activation and steady-state inactivation voltage-dependence at room temperature and following warming of the bath solution. B) Plot of the Ca^2+^ conductance normalized to the maximum g_Ca_ recorded in room temperature conditions shows that g_Ca_ increased approximately 2-fold with warming (n = 7 TC neurons). *p<0.05, **p<0.01. C) Activation and steady-state inactivation plots show that the LVA calcium current activation and steady-state inactivation curves shifted to more hyperpolarized potentials with warming. D & E) Plots of LVA activation and steady-state inactivation V50 values measured with Boltzmann fits at room temperature and following warming of the bathing solution (n = 7 TC neurons).

As noted above, the spiking behavior of dLGN TC neurons is also strongly influenced by the presence of a low voltage-activated (LVA) T-type Ca^2+^ current [15,16,40]. When TC neuron Vrest is more hyperpolarized, this relieves T-type inactivation so that depolarization triggers a LVA Ca^2+^ spike with Na^+^ action potentials riding atop it. In contrast, when TC neuron Vrest is slightly more depolarized, a greater proportion of T-type channels are in an inactivated state and TC neuron spiking becomes more tonic. To test whether temperature modulates T-type current properties in dLGN TC neurons, LVA currents were recorded from a holding potential of -100 mV using voltage-clamp protocols to measure the voltage-dependence of both inactivation and activation (Fig 7). Warming the bath led to a doubling of LVA conductance (2.0 ± 0.3-fold increase; measured at -55 mV; Fig 7A & 7B). The Q10 for the T-type current measured at -55 mV was 2.7 ± 0.53. Warming the bath led to a hyperpolarizing shift of T-type current activation and inactivation curves (Fig 7C). The activation V50 was -67.4 ± 1.2 mV at room temperature and -72.6 ± 1.2 mV in warmed conditions (p = 0.014; n = 7; -5.2 ± 1.5 mV shift; Fig 7D). The inactivation voltage-dependence similarly shifted by -4.3 ± 1.5 mV (-73.8 ± 1.3 mV at room temperature to -78.1 ± 1.2 mV in warmed conditions; p = 0.02; Fig 7E).

**Fig 8:**
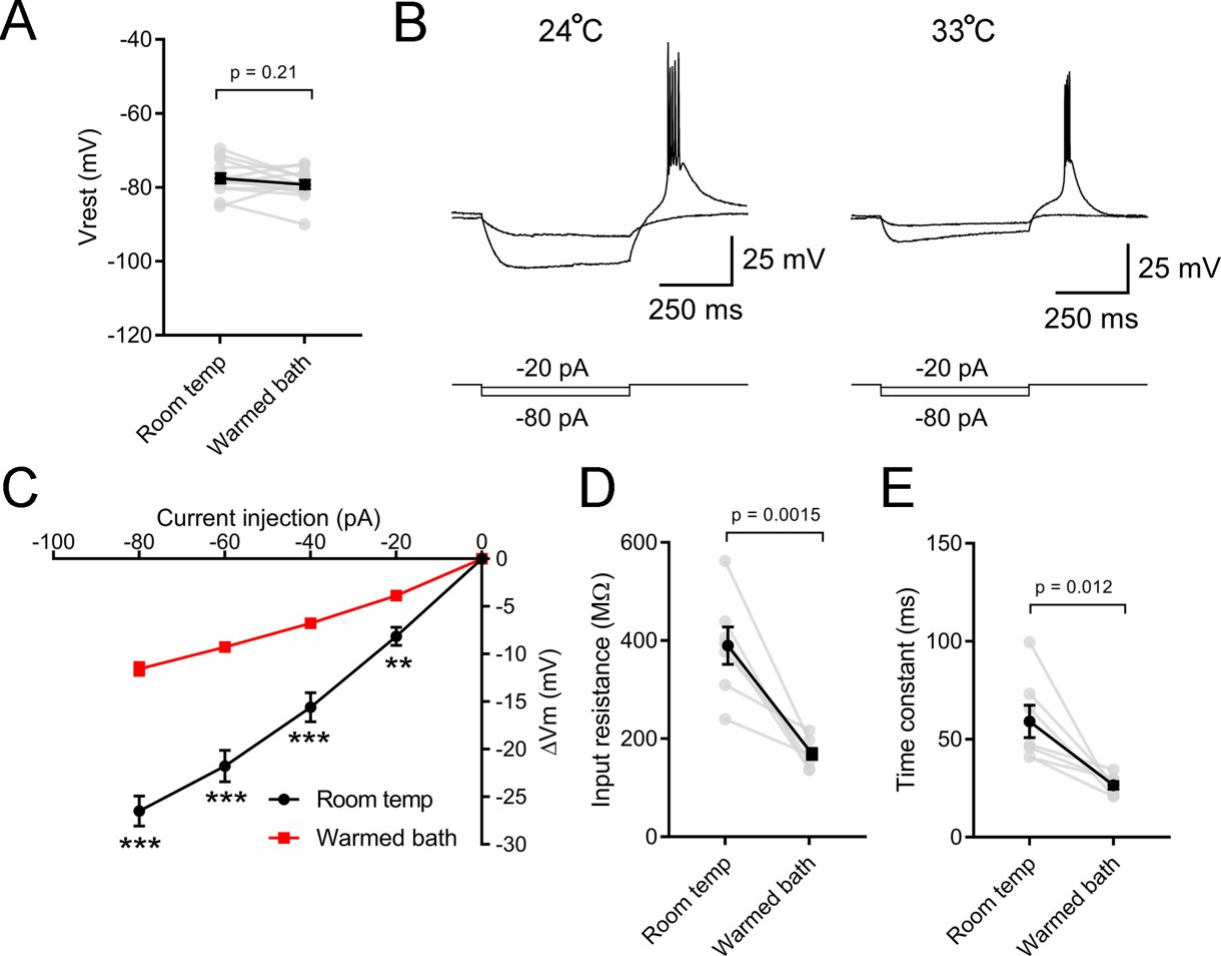
Temperature regulates passive membrane properties of dLGN TC neurons. A) Resting membrane potential was not significantly altered by warming of the bathing solution (n = 13 TC neurons, Wilcoxon matched pairs test). B) Whole-cell Current-clamp recordings from a TC neuron in response to hyperpolarizing current injections at room temperature and following warming of the bathing aCSF. C) Plot of voltage responses to current injections of varying amplitudes at room temperature and after warming the bathing solution shows that warming reduced the voltage-responses. **p<0.01, ***p<0.005. D) Input resistance, measured from a linear fit of the voltage responses to current injections of -20 and -40 mV, was reduced by warming. E) Warming led to an acceleration of the membrane time constant measured with a single exponential fit of the voltage response to a -20 pA current injection.

Changes in spiking behavior can be influenced by resting membrane potential, which could potentially alter the voltage needed to reach spike threshold and change the proportion of inactivated Ca^2+^ and Na^+^ channels. Although increasing bath temperature appeared to cause a small hyperpolarization of TC neuron resting potential (-77.5 ± 1.3 mV in room temperature to -79.2 ± 1.2 mV in warmed conditions; -1.7 ± 1.3 mV change; n = 13; Fig 8A), this was not statistically significant (p = 0.21, Wilcoxon). However, warming the bath did lead to a decrease in TC neuron input resistance and membrane time constant, measured as the slope of a straight line fit to the voltage deflections evoked by -20 pA, and -40 pA current stimuli (Fig 8B–8E); input resistance was reduced from 390 ± 38 MΩ to 169 ± 11 MΩ (p = 0.0015; n = 7) while the membrane time constant was reduced from 59 ± 8 ms to 26 ± 2 ms (p = 0.012; n = 7).

**Fig 9:**
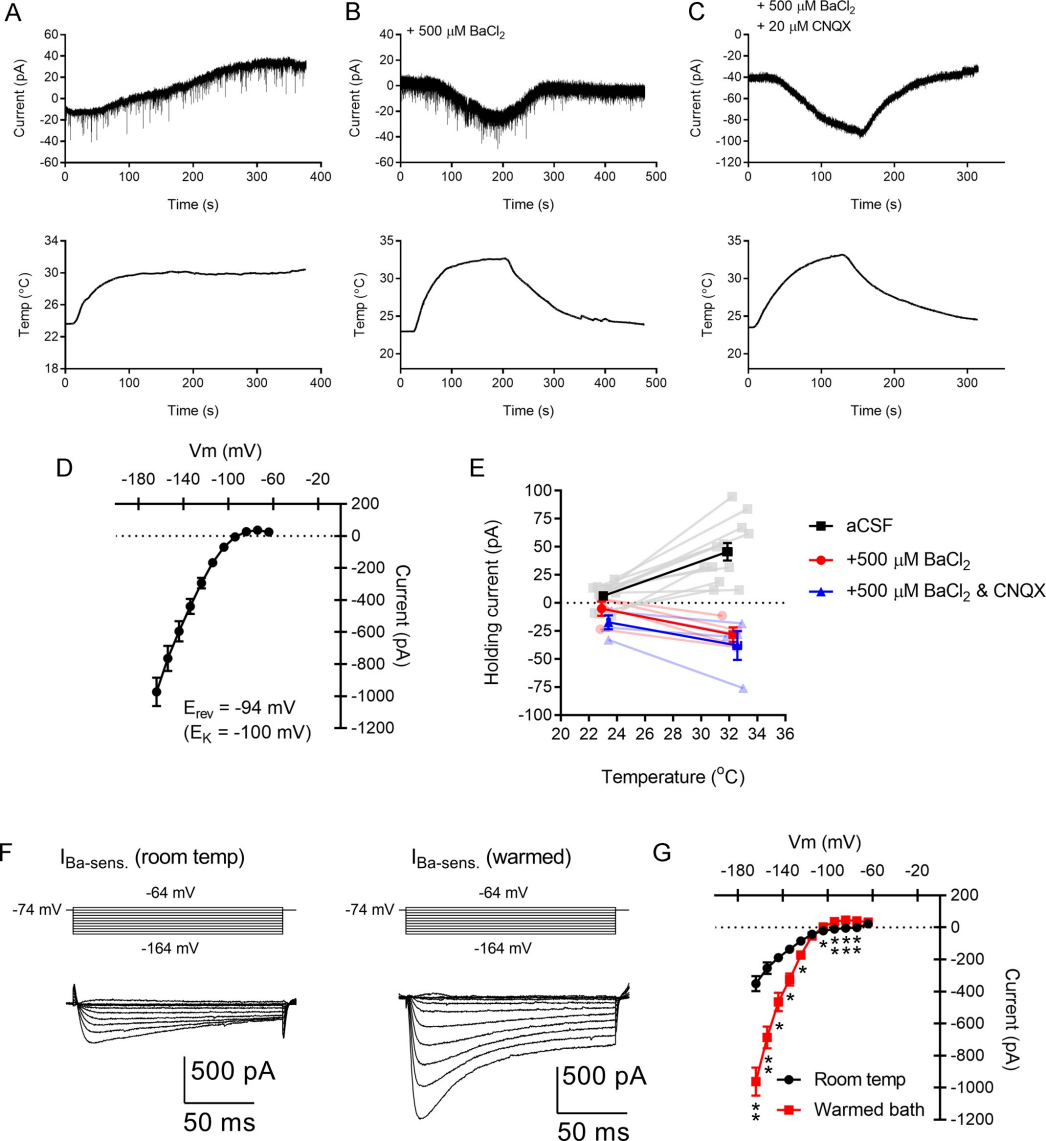
Temperature regulates an inwardly-rectifying potassium current (Kir) in dLGN TC neurons. A) Warming leads to a net outward current recorded from a dLGN TC neuron. B) In a different TC neuron recorded in the presence of 500 μM BaCl_2_, a blocker of Kir, warming instead leads to a slight inward current. The current recovers following return to room temperature aCSF. C) In another TC neuron recorded in the presence of BaCl_2_ and 20 μM CNQX (a blocker of AMPA-type glutamate receptors), warming continues to trigger an inward current. D) Current-voltage plot of the warming-gated current shows that it strongly rectifies and reverses close to the predicted K^+^ Nernst equilibrium potential. E) Plot of holding current against bath temperature for three conditions: aCSF, aCSF + 500 μM BaCl_2_, and aCSF + BaCl_2_ + 20 μM CNQX. The addition of CNQX did not prevent the temperature-triggered inward current. The horizontal error bars are very small and obscured by the markers. F) The Ba^2+^-sensitive current recorded at room temperature and in warmed aCSF from a single TC neuron obtained by subtraction of membrane currents in response to a series of voltage steps recorded in the absence and presence of 500 μM BaCl_2_. G) Plot of the Ba^2+^-sensitive current (presumptive Kir) from 8 TC neurons at room temperature and in warmed conditions showing that its amplitude increased with warming. *p<0.05, **p<0.001.

The origin of this change in input resistance was investigated using voltage-clamp recordings at a holding potential of -74 mV. In these recordings, warming the bath solution from room temperature to ~32°C evoked an outward current of 39 ± 6.7 pA (I_hold_ = +6.2 ± 3.0 pA at room temperature to +45.6 ± 7.9 pA in warmed conditions; p < 0.001; n = 12; Fig 9A–9C). The ionic basis of the warming-evoked membrane current was investigated as the difference current measured from a series of hyperpolarizing and depolarizing voltage steps (-164 to -64 mV, 10 mV increments) recorded at room temperature and in warmed conditions. The heat-activated current showed a strong inward rectification and reversed at -94.5 ± 1.5 mV (Fig 9D). This was close to the calculated K^+^ Nernst equilibrium potential calculated for the combination of aCSF and K-based pipette solution used in this experiment (approx. -100 mV). A small outward current is apparent in the I-V plot of these measurements around the holding potential of -74 mV. These current properties resembled the inwardly-rectifying K^+^ current Kir, which is carried by the Kir family of 2-transmembrane domain K^+^ channels. Kir is blocked by Ba^2+^ ions and, consistent with the outward current being largely the result of activation of Kir at the -74 mV holding potential, no outward current was evoked when the bath contained 500 μM BaCl_2_ (Fig 9B & 9E). Instead, in the presence of Ba^2+^, warming led to a small inward current (23.1 ± 4.6 pA; n = 4; p = 0.02). This heat-activated inward current was not the result of increased AMPA-type glutamate receptor activation, as it was not blocked by 20–40 mM CNQX applied along with Ba^2+^ (20.7 ± 7.9 pA, n = 4; p = 0.80; Fig 9C & 9E). When the Ba^2+^-sensitive components were isolated to determine effects on Kir alone, warming the bath caused an increase in Ba^2+^-sensitive current amplitude (Fig 9F & 9G). When adjusted for driving force and measured at -134 mV, this was a 2.8 ± 0.6-fold increase in Kir conductance (p = 0.0035), which gave a Q10 of 3.0 ± 0.7 (n = 8).

## Discussion

The results of this study show that warming leads to a decrease in spiking responses of thalamocortical relay neurons in the dLGN during optogenetic stimulation of retinal ganglion cell axon terminals. This was associated with an increase in retinogeniculate synaptic vesicle release probability and a complex array of changes in the function of membrane currents responsible for passive membrane properties and spiking behavior including Kir, LVA T-type Ca^2+^ currents, and voltage-gated Na^+^ currents. These data suggest that the combination of increased synaptic depression along with a Kir-associated drop in membrane resistance and acceleration of the membrane time constant overrides warming-triggered enhancement of voltage-gated Na^+^ and T-type Ca^2+^ current activation. As a result, the net effect of warming reduces the ability of retinogeniculate synaptic inputs to trigger TC neuron spiking.

Temperature is a powerful modulator of synaptic transmission, although the precise effects of temperature on presynaptic release properties and post-synaptic receptors appear to vary [34–36,43–48]. In the present study, warming led to an increase in EPSC amplitude that was largely attributable to an increase in Pr assessed with measurements of mEPSC frequency, paired-pulse stimulation experiments, and high-frequency stimulus trains [30]; mEPSC frequency doubled at warmer temperatures, synaptic depression was more pronounced in paired pulse experiments, and train experiments showed an ~1.35-fold increase in Pr. Although retinal ganglion cell inputs to dLGN TC neurons make up only ~10% of synapses, these provide the major “driver” input to the dLGN. A larger number of relatively weak glutamatergic corticogeniculate synapses have a low Pr and modulate TC neuron output [18,49–52]. While paired pulse and train experiments focused exclusively functional measurements on retinogeniculate synapses by virtue of ChR2 reporter expression in retinal neurons [26], the mEPSC measurements represent a combination of inputs from retinogeniculate and corticogeniculate pathways. The increase in mEPSC frequency is consistent with the increased Pr measured using optogenetic stimulation of the retinogeniculate pathway, and might indicate that warming also leads to increased Pr at corticogeniculate synapses as well.

The current study also demonstrates that warming causes a small increase in quantal mEPSC amplitude. This likely contributed to the increased evoked EPSC amplitudes and this might be due to warmer temperatures favoring a higher conductance state of the post-synaptic AMPA receptors [53]. At the calyx of Held and other mammalian synapses, warming has been shown to cause an increase in mEPSC amplitude [34,53,54] while at amphibian hair cell synapses, mEPSC amplitude was unchanged [35].

The effects on Pr in the current study are largely consistent with data from V1 pyramidal neurons, which show that increasing temperature leads to increased synaptic reliability (fewer failures, less synaptic jitter) [45,48]. However, they contrast with a recent study of bullfrog papillar hair cells in which warming had no effect on Pr [35]. Additionally, other studies have shown varying results on the mechanisms behind enhanced synaptic transmission, showing that warming temperatures enhances synaptic vesicle release by increasing the size of the presynaptic vesicle pool and/or increasing the rate of vesicle pool replenishment [34,36,37,43,44,46,55]. In the current study, warming did appear to accelerate synaptic vesicle pool refilling at retinogeniculate synapses (measured as a change in cumulative EPSC slope in train experiments), but this was not accompanied by any increase in vesicle pool size. Enhanced Pr with warmer temperature is likely the result of several combined mechanisms including altered presynaptic action potential shape and kinetics of presynaptic Ca^2+^ domains, altered energetics of the presynaptic SNARE complex and vesicle priming processes, and accelerated endocytic membrane retrieval [56–58].

dLGN TC neurons also receive inhibitory input via local GABAergic interneurons and GABAergic projection neurons in the thalamic reticular nucleus [59]. Inhibitory inputs are driven by excitation from both retina and cortex [59] and temperature-dependent changes in those pathways, in addition to any temperature-dependent changes in interneuron excitability and GABA release processes, are likely to alter inhibition of TC neurons. Since the experiments in this study were conducted in the presence of picrotoxin to block inhibition, these possibilities remain to be tested.

Neuronal spike output depends on the properties of synaptic input (presynaptic release and postsynaptic receptors) and how the post-synaptic neuron integrates those inputs via the combination of passive and active membrane properties to trigger action potentials. Warming the bath solution ultimately had the effect of reducing synaptically-driven TC neuron spiking by concentrating spikes largely to the first stimulus generated from a RGC spike train. The temperature-dependent increase in Pr had the effect of enhancing the first EPSC and causing greater depression of subsequent EPSCs, which is likely a significant contributor to this altered spiking behavior. Warming also led to pronounced changes in spike generation in TC neurons. Most notably, warming led to a reduction in excitability at current injections <360 pA, which is similar to the temperature-dependence of spiking behavior seen in DRG somatosensory neurons [6] and neurons in the trigeminal ganglion [60]. At greater amplitude current injections, however, warming increased TC neuron spiking which is more in keeping with studies pointing toward an increase in excitability at higher temperatures [10,35,61]. Warming also increased the spike frequency adaptation, so that more spikes were concentrated at the beginning of a stimulus.

Despite a small hyperpolarizing trend in dLGN TC neurons (1.7 mV hyperpolarization), warming did not have a statistically significant effect on resting membrane potential, indicating that changes in excitability observed in this study were not simply the result of dramatic changes in Vrest relative to spike threshold. Other studies show a diversity of effects of temperature on Vrest [62]. In dorsal horn and hippocampus, for instance, physiological warming or hyperthermia depolarized neurons [10,61]. In amphibian hair cells, Vrest became slightly more hyperpolarized in whole-cell recordings and did not change in perforated patch recordings following warming [35]. In recordings from locust neurons, warming led to slight hyperpolarization [63].

Despite the diversity of effects on Vrest, increased temperature appears to consistently reduce neuronal input resistance. The results presented above show that TC neuron Rin dropped from approximately 400 MΩ to approximately 200 MΩ when the brain slice was warmed. This surely results from the combined gating of several ion channels near the resting potential. For instance, the increase in presynaptic glutamate release (increased mEPSC frequency) will lead to greater AMPA-type glutamate receptor channel current, which likely makes a contribution here. This was also associated with an acceleration of the membrane time constant, which has the effect of reducing temporal summation of excitatory synaptic inputs. Indeed, accelerated decay of post-synaptic potentials can be seen in the example recordings in Fig 1. Thus, although warming led to an increase in the Pr and consequent increase in amplitude of the evoked post-synaptic currents, this drop in input resistance is likely a major reason why those synaptic inputs failed to evoke more spiking. The data in Fig 1 hint at this, as sub-spike threshold EPSP amplitudes were slightly reduced in warmed conditions.

The cellular mechanisms underlying the change in Rin are likely complex. For instance, the results above show that warming triggered a modest net outward current of approximately 40 pA. Current-voltage analysis revealed that it was likely carried by K^+^ ions and had an inward-rectifying voltage-dependence suggesting it was the result of activation of Kir by warming. Consistent with this, the warming-triggered outward current was blocked by extracellular Ba^2+^, a blocker of Kir channels. Another study of Kir in dopaminergic neurons of the mouse olfactory bulb has shown that Kir is mildly temperature-sensitive, with a Q10 of ~1.22 [64], similar to Kir recorded in cardiac ventricular cells (1.28) [65]. An ATP-sensitive Kir channel was reported to have a Q10 of 1.38 [66] while recordings of another ATP-sensitive K^+^ current in cardiac myocytes had a similar Q10 at the 20–30°C range but a higher Q10 (2.3) at temperatures between 10–20°C. In contrast, the Ba^2+^-sensitive conductance (putative Kir) in dLGN TC neurons had a higher Q10 of ~3.

Interestingly, in the presence of Ba^2+^, the outward current was blocked and warming instead triggered a small inward current. This did not appear to be the result of bulk extracellular glutamate and activation of AMPA receptors, as it was not blocked by CNQX. Thalamic TC neurons express HCN channels and show a modest I_h_ current [67–69]. It appears that I_h_ voltage-dependence is largely unaffected at the temperature range used in this study, although I_h_ current amplitude can be enhanced by warming and previous studies have suggested that I_h_ amplitudes have Q10 of ~3 [8,70–72]. HCN2 channels play an important role in TC neuron resting potential and ~18% of I_h_ is active around a resting potential of -71 mV [73]. Since I_h_ reverses around -20 to -30 mV due to HCN channel’s slight selectivity for K^+^ over Na^+^ [74], enhancing I_h_ conductance at the holding potential used in these experiments (-74 mV) would cause an inward current and, in current-clamp recordings, a depolarizing influence. The slight depolarizing influence of I_h_ might balance the hyperpolarizing influence of the enhanced Kir conductance, possibly explaining why temperature had minimal detected effect on resting membrane potential in TC neurons.

Finally, measurements of voltage-gated Na^+^ and LVA T-type Ca^2+^ currents in dLGN TC neurons revealed that their amplitudes and voltage-dependences were affected by warming the bath. While voltage-gated Na^+^ currents are essential for Na^+^-dependent spiking of TC neurons, LVA T-type Ca^2+^ currents play an important role in TC neuron bursting behavior. LVA Ca^2+^ channel subunits (Ca_V_3.1) have been identified in dLGN TC neurons [2,75] and different T-type subunits appear to be differentially modulated by temperature, affecting both activation and inactivation parameters [1]. Consistent with the major expression of Ca_V_3.1 subunits and reported evidence for temperature sensitivity of Ca_V_3.1 gating from heterologous expression studies [1,75], warming nearly doubled the LVA conductance and led to a leftward (hyperpolarized) shift in the voltage-dependence of LVA current activation and steady-state inactivation curves. Temperature effects on TC neuron LVA current activation and inactivation has been noted previously and had Q10 values of 2.5 [2], similar to the results of the current study. Warming also caused a leftward shift of the Na^+^ activation curve and a rightward shift of the steady-state inactivation curve, although peak amplitude of the Na^+^ current was not significantly altered. Combined, this shift in two of the major voltage-gated currents underlying TC neuron spike generation would serve to provide a larger LVA Ca^2+^ spike at more hyperpolarized voltages, allowing weaker inputs to evoke robust spiking.

The above results show how temperature affects the function of synaptic inputs and intrinsic currents of TC relay neurons in the dLGN to affect TC neuron spike output in response to synaptic input. Additionally, they reinforce the importance of LVA T-type currents in TC neuron spiking behavior and reveal a key role of Kir in setting TC neuron membrane properties. These results resemble those from temperature studies performed in multiple neuron types in different brain nuclei, although they differ from others, highlighting that temperature can affect neuronal function in complicated ways depending on the composition of synaptic inputs and ion channel complement. Additionally, these findings highlight the importance of careful control of temperature in electrophysiological experiments in that temperature differences between preparations can have complex and unpredictable effects on the ways that neurons integrate synaptic inputs to trigger spike outputs.

## Supporting information

S1 Fig Synaptically-driven dLGN thalamocortical (TC) relay neuron spiking is inhibited by warming. Current-clamp traces from each of the six TC neurons in which retinogeniculate inputs were stimulated using a stimulus sequence derived from a retinal ganglion cell spike train (as in Fig 1). Although the spiking responses varied at room temperature, warming reduced overall spiking and concentrated the spiking to the beginning of the stimulus sequence. Spiking recovered after returning to room temperature conditions. 19723cell5 and 19723cell9 in this figure are also shown in Fig 1 in the main manuscript.
(PDF)

S1 Data (XLSX)
