# Supplementary material for: Temperature effects on synaptic transmission and neuronal function in the visual thalamus
Source: PLoS One. 2020 Apr 30;15(4):e0232451. doi: 10.1371/journal.pone.0232451 (PMC7192487; doi:10.1371/journal.pone.0232451)
Supplement: S1 Fig — Current-clamp traces from each of the six TC neurons in which retinogeniculate inputs were stimulated using a stimulus sequence derived from a retinal ganglion cell spike train (as in Fig 1). Although the spiking responses varied at room temperature, warming reduced overall spiking and concentrated the spiking to the beginning of the stimulus sequence. Spiking recovered after returning to room temperature conditions. 19723cell5 and 19723cell9 in this figure are also shown in Fig 1 in the main manuscript. (PDF) [file pone.0232451.s001.pdf]

S1 Figure

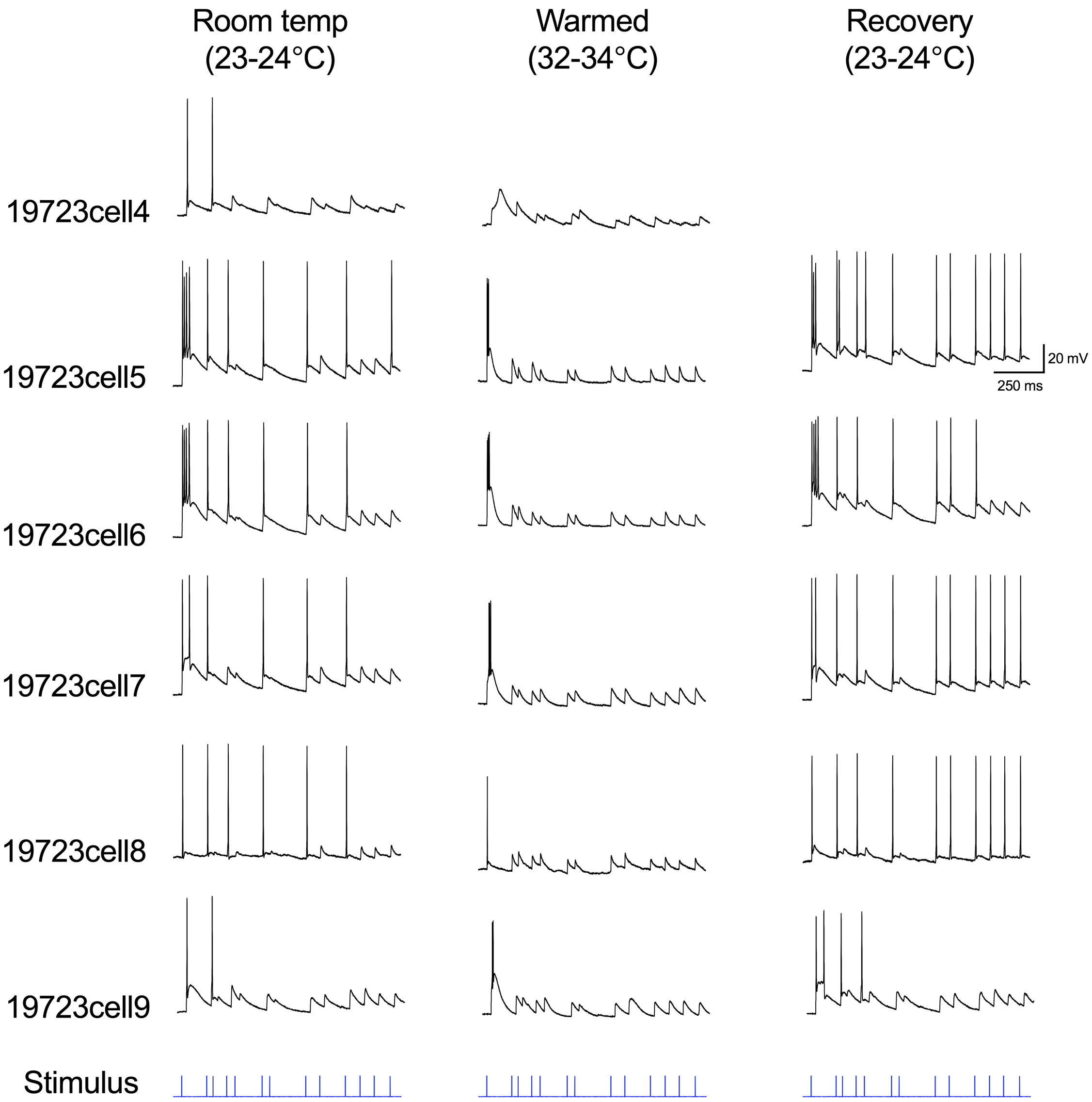

S1 Figure Legend – Synaptically-driven dLGN thalamocortical (TC) relay neuron spiking is inhibited by warming. Current-clamp traces from each of the six TC neurons in which retinogeniculate inputs were stimulated using a stimulus sequence derived from a retinal ganglion cell spike train (as in Figure 1). Although the spiking responses varied at room temperature, warming reduced overall spiking and concentrated the spiking to the beginning of the stimulus sequence. Spiking recovered after returning to room temperature conditions. 19723cell5 and 19723cell9 in this figure are also shown in Figure 1 in the main manuscript.
